# A Low Contact Impedance Medical Flexible Electrode Based on a Pyramid Array Micro-Structure

**DOI:** 10.3390/mi11010057

**Published:** 2020-01-01

**Authors:** Song Wang, Jin Yan, Canlin Zhu, Jialin Yao, Qiusheng Liu, Xing Yang

**Affiliations:** 1 The State Key Laboratory of Precision Measurement Technology and Instruments, Department of Precision Instrument, Tsinghua University, Beijing 100084, China; wangs18@mails.tsinghua.edu.cn (S.W.); 13141317443@163.com (C.Z.); liuqiusheng@tsinghua.edu.cn (Q.L.); 2 School of Materials Science and Engineering, University of Science and Technology Beijing, Beijing 100083, China; 41703112@xs.ustb.edu.cn (J.Y.); yaojialin92@gmail.com (J.Y.)

**Keywords:** flexible electrode, polydimethylsiloxane (PDMS), pyramid array micro-structures, low contact impedance

## Abstract

Flexible electrodes are extensively used to detect signals in electrocardiography, electroencephalography, electro-ophthalmography, and electromyography, among others. These electrodes can also be used in wearable and implantable medical systems. The collected signals directly affect doctors’ diagnoses of patient etiology and are closely associated with patients’ life safety. Electrodes with low contact impedance can acquire good quality signals. Herein, we established a method of arraying pyramidal microstructures on polydimethylsiloxane (PDMS) substrates to increase the contact area of electrodes, and a parylene transitional layer is coated between PDMS substrates and metal membranes to enhance the bonding force, finally reducing the impedance of flexible electrodes. Experimental results demonstrated that the proposed methods were effective. The contact area of the fabricated electrode increased by 18.15% per unit area, and the contact impedance at 20 Hz to 1 kHz scanning frequency ranged from 23 to 8 kΩ, which was always smaller than that of a commercial electrode. Overall, these results indicated the excellent performance of the fabricated electrode given its low contact impedance and good biocompatibility. This study can also serve as a reference for further electrode research and application in wearable and implantable medical systems.

## 1. Introduction

Electrodes are important components of implantable or wearable medical monitoring systems. They are used to collect electrocardiography (ECG) and electroencephalogram (EEG) signals. If the collected signals are inaccurate, patient treatment may be delayed, their conditions worsened, or their lives endangered. Therefore, high-quality electrodes are significant for precisely monitoring the physiological parameters of the human body. At present, ECG, EEG, electromyogram (EMG), or electro-ophthalmogram (EOG) signals are measured on a standard silver/silver chloride (Ag/AgCl) wet electrode for medical diagnosis [1]. Such electrodes usually reduce the contact impedance between the skin and electrode by using conductive gels [1]. Standard Ag/AgCl wet electrodes enable low electrode–skin contact impedance and the acquisition of signals with high stability and repeatability. However, the conductive gel may cause problems such as becoming dry with time, which increases the contact impedance and aggravates measurement errors [2]. Conductive gels can also sometimes cause skin irritations or allergic rashes [3,4].

The above problems have prompted researchers to explore dry electrodes with other conductive materials [4,5] and the use of microelectromechanical system technology [6]. For example, Fei Yu et al. (2012) prepared a flexible dry electrode based on parylene substrate to collect ECG signals in zebrafish; their electrode exhibits a good signal-to-noise ratio [7]. Electrode impedance is a criterion for evaluating electrode performance. A smaller impedance corresponds to better collected-signal quality [8]. Some progress has been made in the study of dry electrodes, but some challenges remain, such as those related to reducing the impedance of flexible dry electrodes. To achieve such reduction and obtain high-quality signals, this study theoretically analyzed the impedance of electrodes and the electrode–skin contact impedance. We then proposed a method of reducing electrode impedance by fabricating pyramidal array microstructures on polydimethylsiloxane (PDMS) substrate and adding a parylene transition layer between PDMS and metal film. Finally, the impedance of the flexible electrode was experimentally tested and analyzed.

## 2. Theoretical Analysis of Flexible Electrode Based on Micro-structure

### 2.1. Theoretical Analysis of Selecting Material of Flexible Electrode Substrate

To reduce electrode impedance and enhance the bonding force between substrate and metal film, we selected substrate materials for flexible electrodes on the basis of two aspects: one is the bonding degree between substrate and biological tissues, and the other is flexibility that should be similar to that of biological tissues. The first basis for substrate selection considers the degree of adherence between the substrate and biological tissues. A material should have flexibility similar to that of biological tissues, i.e., they should be easily bent and stretched. The second basis for substrate selection considers the flexibility, i.e., polymer materials with good elasticity should be selected. Young’s modulus of PDMS in polymer (0.4–1.0 MPa) is much smaller than that of other polymer materials, such as parylene (2400–3200 MPa). Thus, PDMS is easier to bend and deform and is more suitable for the fabrication and bonding of flexible substrates with large thickness. At the same time, PDMS is inexpensive and simple to manufacture; it can be prepared by simple spin coating. These characteristics indicate the suitability of PDMS materials as the substrate material for flexible electrodes. However, considering the impedance stability and thermal expansion between substrate and metal, two major problems arise in selecting PDMS material as substrate to directly bond with metal. On one hand, a porous PDMS material has strong permeability, and an electrode easily absorbs water owing to capillary action, which can lead to changes in electrode impedance. On the other hand, the weak adhesion forces between PDMS and metal confer difficulty in the deposition and transfer of a metal electrode onto PDMS [9]. Considering that the thermal-expansion coefficient of PDMS (α_PDMS_ ≈ 20 × 10^−5^ K) relatively differs from that of gold (α_Au_ ≈ 1.42 × 10^−5^ K) [10], the metal film on PDMS substrate during electrode fabrication and storage is prone to cracking because of changes in temperature. Consequently, electrode impedance increases.

To address the two problems, a layer of parylene can be introduced between PDMS substrate and gold electrode. On one hand, the water permeability of parylene is very low, so water cannot easily penetrate parylene. The combination of parylene and PDMS can offset the shortcomings of the water absorption of PDMS and overcome the change in electrode impedance. On the other hand, parylene is also a common material for preparing flexible substrates. Its thermal-expansion coefficient (α_parylene_ ≈ 3.5 × 10^−5^ K) is similar to that of gold [10], and it is hardly affected by cracks caused by temperature factors after bonding with gold. The resulting PDMS–parylene layer possesses a more favorable combination of stiffness and flexibility that can improve film manageability, and the fabrication process is straightforward [11]. This process overcomes the disadvantages of the commonly used PDMS flexible electrode, which induces impedance changes owing to its easy water absorption and crack and wrinkle formation.

### 2.2. Theoretical Analysis of Contact Impedance of Flexible Electrode–skin

**Figure 1:**
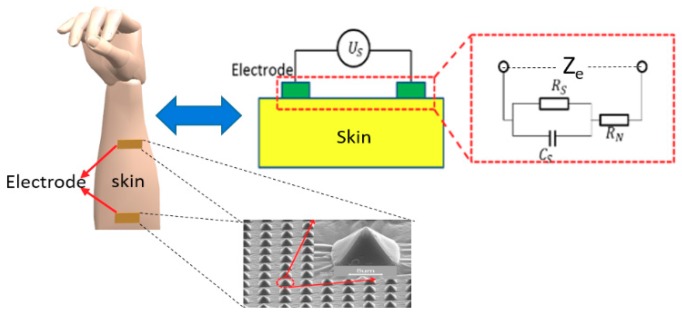
Skin–electrode contact impedance equivalent circuit diagram.

To analyze electrode–skin contact impedance, selecting a suitable impedance circuit model is very important. We utilize the H. Feriberger equivalent circuit model, which is extensively used in medicine [12], as is shown in the following Figure 1:

Where $R_{c}$, $R_{s}$, $R_{N}$, and $Z_{e}$ are the impedance of capacitance, impedance of electrode - skin, internal fixed impedance of human body (a constant value) [12] and the total impedance(a series–parallel structure of skin impedance, skin capacitance, and internal impedance of human body). $u_{s}$ is the given constant voltage, $C_{s}$ is the skin capacitance between two electrodes. $\varepsilon_{0}$ and $\varepsilon_{r}$ are the vacuum dielectric constant and skin dielectric constant, respectively. A is the contact area of flexible electrodes with the skin, d is the distance between flexible electrodes, ρ is the skin impedance coefficient, ω is the angular frequency. The related formulas are as follows:

(1) $$C_{s}=\frac{\varepsilon_{0}\times \varepsilon_{r}\times A}{d}$$

(2) $$R_{s}=\rho \times \frac{d}{A}$$

(3) $$R_{c}=\frac{1}{j\omega \times C_{s}}=\frac{d}{j\omega \times \varepsilon_{0}\times \varepsilon_{r}\times A}$$

(4) $$R_{c}//R_{s}=\frac{\frac{\rho d}{A}\times \frac{d}{j\omega \varepsilon_{0}\varepsilon_{r}A}}{\frac{\rho d}{A}+\frac{d}{j\omega \varepsilon_{0}\varepsilon_{r}A}}=\frac{\rho d}{A(1+j\omega \varepsilon_{0}\varepsilon_{r}\rho )}$$

(5) $$Z_{e}=R_{c}//R_{s}+R_{N}=\frac{R_{s}}{1+j\omega R_{s}C_{s}}+R_{N}=\frac{\rho d}{A\left(1+j\omega \varepsilon_{0}\varepsilon_{r}\rho \right)}+R_{N}$$

**Figure 2:**
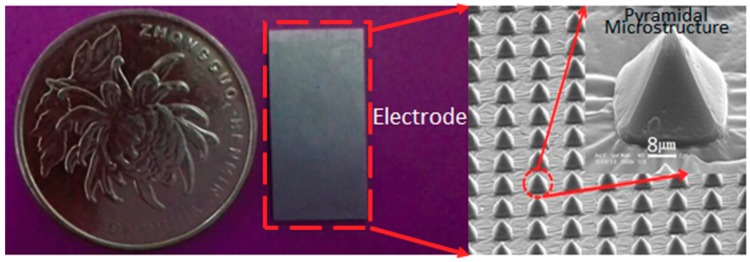
The SEM photographs of pyramidal microstructure arrays.

As previously mentioned and analyzed, a smaller electrode–skin contact impedance corresponds to a higher quality of collected signals. Thus, the contact impedance directly affects electrode performance. According to Equations (2) and (3), a larger contact area of electrodes with the skin means a smaller impedance of the electrode–skin contact capacitance. Moreover, the total electrode–skin contact impedance decreases according to Equations (4) and (5), so increasing the contact area between the electrode and skin can produce high-quality signals. To increase the contact area with skin per unit area, some special microstructures were designed and fabricated on the electrode contact surface. These microstructures were micro-pyramidal arrays with a base of 8 μm × 8 μm and an 8 μm interval between each pyramidal microstructure. This dense pyramidal array effectively increased the contact area with skin per unit area without increasing the electrode size. The images of the pyramidal microstructures are shown in Figure 2.

In this study, 781,250 microstructural pyramids were fabricated on the surface of PDMS flexible dry electrode with dimensions of 2 cm (L) × 1 cm (W). The total surface area of the flexible dry electrode with micro-pyramidal array was 2,363,072 μm^2^, which was 363,072 μm^2^ larger than that of the planar flexible dry electrode. Therefore, the contact area of the flexible dry electrode can be increased by 18.15% through the fabrication of pyramidal microstructures on PDMS substrates, thereby effectively reducing the contact impedance between electrode and skin.

## 3. Fabrication of Flexible Electrodes

The fabrication of flexible electrodes based on PDMS pyramidal arrays was divided into many steps. Firstly, a mold of pyramidal array pits was fabricated by etching on silicon wafers. The etching method was as follows. Apply reactive ion etching (RIE) technology to etch silicon nitride with a thickness of 300 nm on the upper surface of silicon wafer for 30 min. Then use the anisotropic wet etching method of silicon: the silicon wafer was put into 33.3% potassium hydroxide solution for etching, while heating in water bath at 80 °C for 10 min.

Secondly, fabricate PDMS flexible substrate with pyramid microstructure. The method was as follows. Put the silicon template with pyramid microstructure into acetone, ethanol, deionized water and mold release agent in turn and perform ultrasonic treatment for 10 min respectively. Then take out the silicon wafer and dry it to obtain the pretreated silicon template. At the same time, we mixed the PDMS liquid elastomer and curing agent uniformly with the mass ratio of 10:1 and placed the mixture in a vacuum chamber to be pumped for 15 min so that bubbles in the mixture could be fully discharged. After that, pour the PDMS mixture on the pretreated silicon template with pyramid microstructure. At the same time, spin-coat the PDMS mixture uniformly with a spin coater. Next, place the silicon wafer with PDMS mixture in an oven for baking at 70 degree for 2 h to solidify the PDMS mixture. 

**Figure 3:**
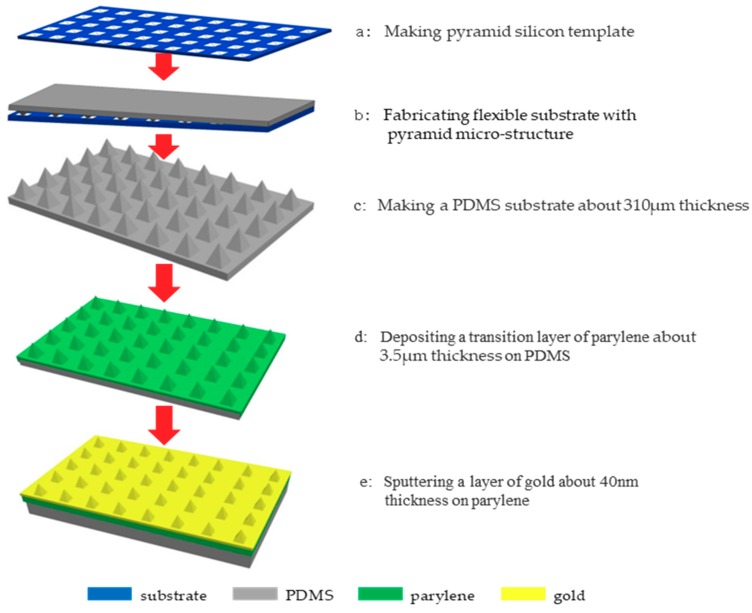
Flow chart of flexible electrode fabrication.

After solidification, strip the solidified PDMS from the silicon wafer to obtain a 310 μm thick PDMS flexible substrate with pyramid microstructure, and then a 3.5-μm-thick parylene-C layer was fabricated on the prepared PDMS substrates by chemical vapor deposition. Finally, a metal film was magnetron sputtered onto parylene-C. In this study, gold was selected as the metal layer material with 40 nm thickness. This fabrication was simple and inexpensive as the fabrication of PDMS film substrate did not require any special deposition equipment. The fabricated PDMS film was also very flexible. The detailed fabrication process of flexible electrodes is shown in Figure 3.

## 4. Testing Performance of Electrode

### 4.1. Electrode Self-Impedance Testing

In this experiment, two types of PDMS flexible electrodes with and without a parylene transition layer were compared and analyzed. The impedance of the electrode was tested at 20 Hz. The impedance of PDMS pyramidal array electrode without the parylene transition layer was 74.94 MΩ, and that of PDMS pyramidal array electrode with the parylene transition layer was 49.52 Ω. After measuring the electrode impedance, we also observed the morphology of the two electrodes under a microscope, and the results were as follows.

**Figure 4:**
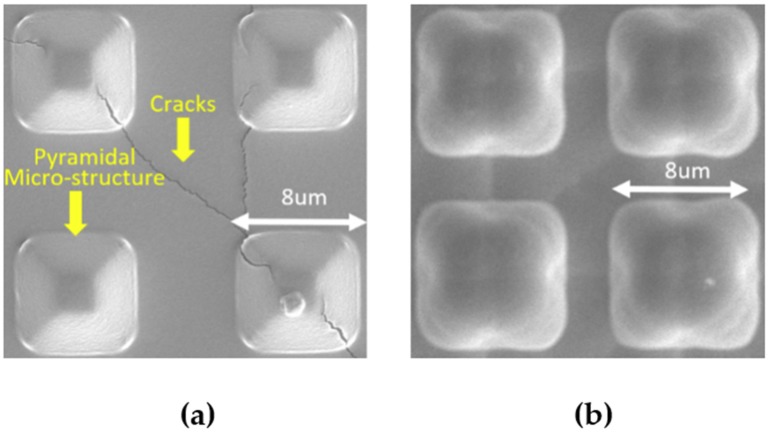
Scanning electron microscope (SEM) photographs of metal films on electrodes (**a**) Electrodes without parylene transition layer (**b**) Electrodes containing the parylene transition layer.

According to Figure 4, several cracks were present on the metal film of the electrode without the parylene transition layer, and some of the micro-pyramid structures collapsed and cracked. Meanwhile, the contact surface morphology of the PDMS electrode with the parylene transition layer was intact, barely producing micro-cracks. This finding was due to the thermal-expansion coefficient of PDMS greatly differing from that of gold, which caused the destruction of gold on the pyramid structure when the metal was directly sputtered. The thermal-expansion coefficient of parylene is close to that of gold, but pyramid deformation can hardly be transmitted and did not affect the gold film during magnetron sputtering after the parylene transition layer was added. The experiments demonstrated that adding the parylene transition layer between PDMS and gold can effectively prevent the breakage of the metal film, thereby ensuring the small and stable impedance of PDMS electrode with pyramidal array microstructure.

### 4.2. Characterization and Testing Performance of Electrode–Skin Contact Impedance

To verify whether the fabricated flexible electrode based on PDMS pyramidal array microstructure had low electrode–skin contact impedance, we used a commercial electrode (Tianrun Sunshine (Beijing) Commercial Products Co., Ltd. Beijing, China) with the same size to perform comparative experiments. For impedance analysis, a Precision Impedance Analyzer (Wayne Kerr 6500B, made in UK, Wayne Kerr Electronics, West Sussex, UK) was used. We obtained the spectrum of the electrode–skin contact impedance for flexible and commercial electrodes separately by scanning the frequency, whose interval was set from 20 Hz to 1 kHz. The driving voltage was set at 1 V. The two electrodes were attached to the left arm of a test person, and their center distance was kept at 10 cm. The experimental data were analyzed using MATLAB (R2017b, The MathWorks, Inc., Natick, MA, USA).

**Figure 5:**
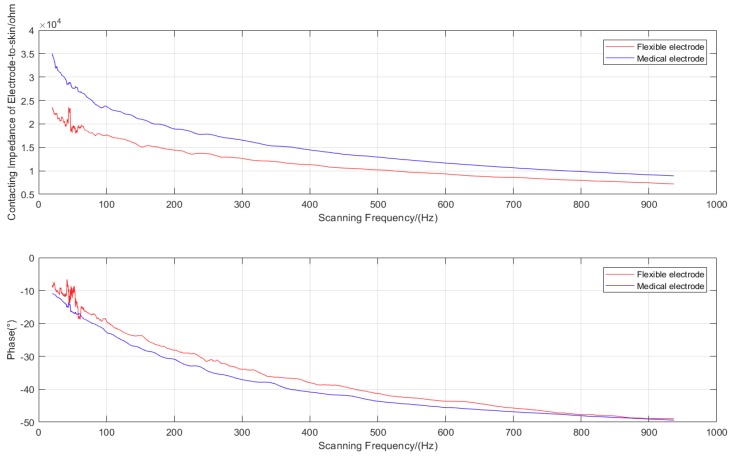
Comparison of skin contact impedance between the commercial electrodes and the flexible electrodes with pyramidal array micro-structure.

Figure 5 shows that the difference between the fabricated flexible electrode and commercial electrode was obvious at low frequencies; both their contact impedance and phase gradually decreased with increased scanning frequency. We found that although the scanning frequency was affected by power-frequency interference at around 50 Hz, the impedance value of the fabricated flexible electrode at any frequency was always smaller than that of the commercial electrode, indicating its superiority over the commercial electrode.

## 5. Conclusions

This study proposed a simple and efficient method of fabricating a low-contact-impedance and flexible electrode with pyramid array microstructure. By fabricating the micropyramid array on the electrode surface, the contact area of the fabricated electrode with the skin increased, which indirectly reduced the impedance of flexible electrodes. It is shown that the contact area of fabricated electrodes is increased by 18.15% per unit area with adding pyramid array micro-structure on the surface of the electrode through calculations.

Upon analysis of the contact impedance of the flexible electrode–skin circuit equivalent model, we studied the simple process of fabricating the flexible electrode with pyramid array microstructure. Experimental results demonstrated that the contact impedance ranged from 23 to 8 kΩ at 20 Hz to 1 kHz scanning frequency, which was always smaller than that of the commercial electrode. Therefore, the proposed method is effective and the fabricated flexible dry electrode has good performance in terms of low impedance and good biocompatibility, indicating the potential applications of the electrode in EOG, ECG, and EMG, among others.

## References

[1] Chlaihawi A.A., Narakathu B.B., Emamian S., Bazuin B.J., Atashbar M.Z. (2018). Development of printed and flexible dry ECG electrodes. Sens. Bio-Sens. Res..

[2] Xu S., Dai M., Xu C., Chen C., Tang M., Shi X., Dong X. (2011). Performance evaluation of five types of Ag/AgCl bio-electrodes for cerebral electrical impedance tomography. Ann. Biomed. Eng..

[3] Yokus M.A., Jur J.S. (2015). Fabric-based wearable dry electrodes for bodycontact surface bio-potential recording. IEEE Trans. Biomed. Eng..

[4] Myers A., Huang H., Zhu Y. (2015). Wearable silver nanowire dry electrodes for electro-physiological sensing. Roy. Soc. Chem..

[5] Zhou Y., Ding X., Zhang J., Duan Y., Hu J., Yang X. (2014). Fabrication of conductive fabric as textile electrode for ECG monitoring. Fibers Polym..

[6] Jung H., Moon J., Baek D., Lee J., Choi Y., Hong J., Lee S. (2012). CNT/PDMS composite flexible dry electrodes for long-term ECG monitoring. IEEE Trans. Biomed. Eng..

[7] Yu F., Zhao Y., Gu J., Quigley K.L., Chi N.C., Tai Y.C., Hsiai T.K. (2012). Flexible microelectrode arrays to interface epicardial electrical signals with intracardial calciμm transients in zebrafish hearts. Biomed. Microdevices.

[8] Ying M. (2015). Research on Key Technologies of Intelligent ECG Monitoring System Based on Flexible MEMS Dry Electrode Array. Ph.D. Thesis.

[9] Koh D., Wang A., Schneider P., Bosinski B., Oh K. (2017). Introduction of a Chemical-Free Metal PDMS Thermal Bonding for Fabrication of FlexibleElectrode by Metal Transfer onto PDMS. Micromachines.

[10] Chou N., Yoo S., Kim S. (2013). A Largely Deformablecontact surface Type Neural Electrode Array Based on PDMS. IEEE Trans. Neural Syst. Rehabil. Eng..

[11] Ochoa M., Wei P., Wolley A.J., Otto K.J., Ziaie B. (2013). A Hybrid PDMS-Parylene Subdural Multi-Electrode Array. Biomed. Microdevices.

[12] Yuan G. (2006). Research and Application of Human Body Effect of Low Frequency Current. Master’s Thesis.

